# New Insights Into the Mechanisms and Biological Roles of D-Amino Acids in Complex Eco-Systems

**DOI:** 10.3389/fmicb.2018.00683

**Published:** 2018-04-06

**Authors:** Alena Aliashkevich, Laura Alvarez, Felipe Cava

**Affiliations:** The Laboratory for Molecular Infection Medicine Sweden (MIMS), Department of Molecular Biology, Umeå University, Umeå, Sweden

**Keywords:** D-amino acids, D-methionine, D-arginine, bacteria, cell wall, *Vibrio cholerae*

## Abstract

In the environment bacteria share their habitat with a great diversity of organisms, from microbes to humans, animals and plants. In these complex communities, the production of extracellular effectors is a common strategy to control the biodiversity by interfering with the growth and/or viability of nearby microbes. One of such effectors relies on the production and release of extracellular D-amino acids which regulate diverse cellular processes such as cell wall biogenesis, biofilm integrity, and spore germination. Non-canonical D-amino acids are mainly produced by broad spectrum racemases (Bsr). Bsr’s promiscuity allows it to generate high concentrations of D-amino acids in environments with variable compositions of L-amino acids. However, it was not clear until recent whether these molecules exhibit divergent functions. Here we review the distinctive biological roles of D-amino acids, their mechanisms of action and their modulatory properties of the biodiversity of complex eco-systems.

## Introduction

Amino acids have an α-carbon that is connected to four functional groups: an amine group (-NH_2_), a carboxyl group (-COOH), a hydrogen (-H) and a side chain (-R). Therefore, the α-carbon is a stereocenter (or chiral center) of the molecule since depending on the spatial arrangement of these four different groups, two stereoisomers exist: the levorotatory (L) and the dextrorotatory (D). These stereoisomers are not superimposable mirror images to each other. Only in the particular case of glycine, there is a hydrogen atom as side chain -R, therefore glycine does not have a chiral center.

L-Amino acids are essential for life since they provide the building blocks of proteins in all kingdoms of life. D-Amino acids (mainly D-alanine and D-glutamic acid) are also fundamental in microbial physiology where they are key constituents of the peptidoglycan (PG) (Park and Strominger, 1957; Hancock, 1960), an essential part of the bacterial cell wall. The presence of D-amino acids in the peptide moieties of the PG of bacteria makes the cell wall invulnerable to most proteases designed to cleave between L-amino acids. Additionally, the presence of alternative D-amino acids like D-Asp (Bellais et al., 2006; Veiga et al., 2006) or D-Ser at the terminal position of the stem peptide provides tolerance to certain bactericidal agents such as vancomycin (Sieradzki and Tomasz, 1996; Grohs et al., 2000; De Jonge et al., 2002; Reynolds and Courvalin, 2005). Moreover, Lam et al. (2009) reported that diverse bacterial species produce and release to the environment different sets of D-amino acids (non-canonical D-amino acids or NCDAAs) in millimolar range concentration. In *Vibrio cholerae*, the production of D-amino acids in its stationary phase and their incorporation into the PG polymer control the strength and amount of this structure, thereby providing fitness against low osmolarity and stationary phase stresses such as starvation, growth arrest or accumulation of secondary metabolites (Lam et al., 2009; Cava et al., 2011a).

Since this breakthrough, NCDAAs have been recognized as a new type of bacterial effectors, produced by diverse species, and whose biological roles are far from being fully defined. In fact, D-amino acids not only govern PG chemistry, density and strength in D-amino acid-producing and non-producing bacteria (Cava et al., 2011a), but also regulate spore germination and biofilm dispersal in certain species (Hills, 1949; Bucher et al., 2015). D-Amino acids are produced by both highly specific and broad spectrum racemases (Bsr) in bacteria (reviewed in detail by Hernández and Cava, 2016). Conversely to monospecific racemases, Bsr are able to produce D-amino acids from a wide range of both proteinogenic and non-proteinogenic L-amino acids (Espaillat et al., 2014). Bsr-containing bacteria are, in general, Gram-negative bacteria associated to various environments like soil, water or animal hosts. Availability and identity of L-amino acids in those environments would affect the final composition and amount of the D-amino acids.

**FIGURE 1:**
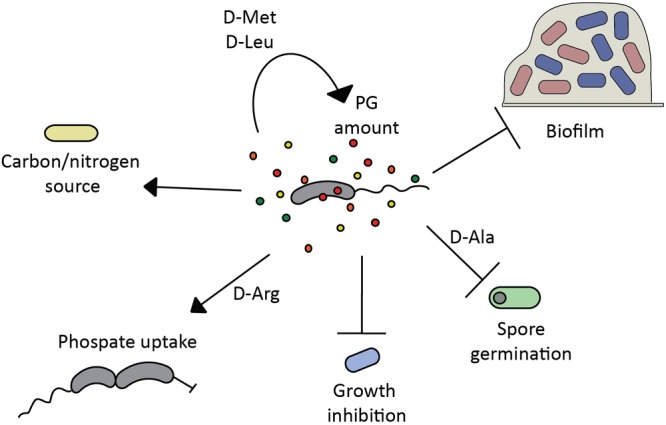
Modulatory properties of D-amino acids in microbial communities. Bacterial D-amino acid production regulates (→) and/or inhibits (T) diverse cellular processes in the producer or other bacteria in the same niche, playing a key role in biofilm formation/disassembly (Rohde et al., 2005; Hochbaum et al., 2011; Ramon-Perez et al., 2014; Rumbo et al., 2016; Yu et al., 2016), spore germination (Hills, 1949), growth (Alvarez et al., 2018), phosphate uptake (Alvarez et al., 2018), peptidoglycan (PG) homeostasis (Lam et al., 2009; Cava et al., 2011a), and can be used as nutrient source (Pikuta et al., 2016).

In this review, we focus on the molecular mechanisms and the ecological consequences that the environmental release of D-amino acids causes in microbial communities and the host (**Figure 1**).

## Role of D-Amino Acids In Microbial Communities

### Biofilm Dispersal by D-Amino Acids

In the environment, bacteria exist in planktonic and biofilm state. Biofilms are bacterial communities held together by a self-produced extracellular polymeric substance (EPS), which is typically composed of protein, exopolysaccharide and often extracellular DNA (Branda et al., 2005; Flemming and Wingender, 2010). In the biofilm, bacteria are effectively protected from harmful environmental threats, and persister cells can develop upon antibiotic attack. These cells can re-emerge once the environment becomes more favorable thereby contributing to chronic infections (Lam et al., 1980; Costerton, 1999; Singh et al., 2000; Post, 2001) and making eradication of biofilms a serious health care issue (Lewis, 2001). Therefore, interfering with biofilm formation or stimulating its dissociation is an attractive strategy to combat bacterial infections and preventing their chronic development. However, biofilms are not just a major problem in clinics, but also in agriculture because of plant loss due to bacterial diseases (Ramey et al., 2004; Matthysse et al., 2005; Koczan et al., 2009; Malamud et al., 2012; Velmourougane et al., 2017), and in industrial water, gas and oil systems, where microbial-induced corrosion causes pipe leakage (Wang et al., 2013; Ramírez et al., 2016; Di Gregorio et al., 2017).

In 2010, Kolodkin-Gal et al. (2010) reported that a mixture of D-amino acids (D-Leu, D-Met, D-Tyr, D-Trp) at nanomolar concentrations could prevent biofilm formation and trigger disassembly of already existing biofilms in *Bacillus subtilis*. Initially, this effect was reported to be due to D-amino acid incorporation in the cell wall, which interfered with the proper localization of TapA (TasA anchoring/assembly protein), leading to the detachment of cell-anchored TasA amyloid fibers, the main structural component of the fibrous biofilm’s scaffold produced by *B. subtilis* (Kolodkin-Gal et al., 2010; Romero et al., 2011). However, later it was found that the *B. subtilis* strain used in this study had a mutation in the *dtd* gene, the D-tyrosyl-tRNA deacylase that makes proteins refractive to D-amino acids’ incorporation (Leiman et al., 2013). Complementation with the wild-type Dtd enzyme made the *B. subtilis* resistant to the biofilm dissociating activity of D-amino acids and thus, Kolodkin-Gal et al. (2010) article has raised a great interest and controversy regarding if and how D-amino acids can influence biofilm stability in different bacteria. For example, Kao et al. (2017) showed that *Pseudomonas aeruginosa* PAO1 biofilm formation is not inhibited by D-Trp (10 mM) and D-Tyr (10 and 1 mM), while Rumbo et al. (2016) reported biofilm inhibition in the same strain by 4 mM D-Trp (10% biofilm reduction) and 4 mM D-Tyr (16% biofilm reduction) using similar methodologies.

A similar situation was observed for *Staphylococcus aureus*. Hochbaum and colleagues found that *S. aureus* SC01 biofilm formation was efficiently inhibited by 500 μM of either D-Tyr, D-Pro or D-Phe, while a mixture of these three D-amino acids was already effective at less than 100 μM (Hochbaum et al., 2011). D-Amino acids did not prevent the initial attachment of the bacterial cells to the surface, but inhibited subsequent growth of the initial microcolonies into larger assemblies by affecting the protein component of the EPS. Production and localization of exopolysaccharide was not significantly affected. The D-amino acid mixture was also able to disassemble already existing *S. aureus* biofilms, but at much higher concentration (10 mM). On the contrary, Sarkar and Pires (2015) reported that *S. aureus* SC01 biofilm formation was not inhibited by D-Tyr or D-Tyr/D-Pro/D-Phe mix even though the authors used millimolar concentrations in the study.

A similar mechanism of biofilm disassembly as in *B. subtilis* has been suggested for *Staphylococcus epidermidis*. The biofilm of *S. epidermidis* contains polysaccharides and proteins such as Aap, which has a PG binding motif and undergoes polymerization to form fibers (Rohde et al., 2005). The authors hypothesize that the polymerization ability of Aap is affected by D-amino acids, which ultimately leads to biofilm disassembly. Different sensitivity to D-amino acids during biofilm formation has been demonstrated for a wide set of pathogenic and non-pathogenic *S. epidermidis* strains (Ramon-Perez et al., 2014). For some strains, biofilm formation was reduced by all D-amino acids tested (D-Leu, D-Tyr, D-Pro, D-Phe, D-Met, and D-Ala), while only some specific D-amino acids or none had an inhibitory effect in other strains. D-Met was the most efficient to inhibit biofilm formation, followed by D-Phe.

Inconsistencies in the activity of D-amino acids as biofilm disassembly agents and variations in the active concentrations were addressed in a methodological paper of Kolodkin-Gal group (Bucher et al., 2016), which showed that biofilm dissociation by D-amino acids is highly dependent on the experimental set-up. The medium used for the pre-culture (rich/defined), the growth phase (logarithmic/stationary), the inoculation ratio and the removal of spent medium before the inoculation are the major factors that account for the variations in the concentration of D-amino acid required to inhibit biofilm development (Bucher et al., 2016).

### D-Amino Acids Target Distinctive Cellular Pathways in Bacteria

In an attempt to categorize the effect of D-amino acids on bacteria, Yu et al. (2016) tested a range of D-Tyr concentrations on the Gram-negative bacterium *P. aeruginosa* and the Gram-positive *B. subtilis*. D-Tyr inhibited biofilm formation in both bacteria at both low, sublethal, concentrations of 5 nM and higher concentrations of 200 μM, while having no effect in intermediate concentrations (1–10 μM). D-Tyr had opposite impact on the EPS production in the two studied bacteria. In *P. aeruginosa*, the level of extracellular protein went down, while it increased in *B. subtilis*. Exopolysaccharide production in *P. aeruginosa* was higher at low concentration of D-Tyr, and decreased at high concentrations, while no change was observed in *B. subtilis*. These results suggest that distinct mechanisms might be involved at different D-Tyr concentrations and they might be species specific.

A systematic approach to test the differential activity of D-amino acids was taken by Rumbo et al. (2016) who evaluated the activity of 18 different D-amino acids on the pathogens *Acinetobacter baumannii* and *P. aeruginosa*. Some D-amino acids inhibited bacterial growth, biofilm formation and adherence to eukaryotic cells, as well as protected alveolar cells from *P. aeruginosa* infection. However, even though some protective effect was observed in mice, the difference in survival of treated and non-treated groups was not statistically significant. In addition, some of the D-amino acids tested affected bacterial growth suggesting an indirect effect in biofilm formation. Overall, the study proposes a bacteria-specific effect of D-amino acids, however, no mechanistic/genetic insights have been provided by the study.

Recently, Alvarez et al. reported that *V. cholerae* produces and secretes high amounts of D-Arg (0.7 mM D-Arg) to the extracellular medium in stationary phase in addition to previously identified D-Met and D-Leu (Lam et al., 2009; Alvarez et al., 2018). Previous screenings failed to identify D-Arg in the stationary phase supernatant, since they relied on the rod-to-sphere morphological transition induced by the supernatant active fractions in a cell wall sensitive mutant (*mrcA*) (Lam et al., 2009). Like in the case of D-Met and D-Leu, D-Arg was produced by *V. cholerae*’s broad spectrum racemase BsrV. However, D-Arg inhibited a wider range of phylogenetically diverse bacteria than any other D-amino acid tested in the study (D-Ala, D-Met, D-Ser, D-His, D-Gln, and D-Phe). Biochemical analysis of PG, microscopy and transposon sequencing revealed that in contrast to D-Met, which is a known modulator of cell wall biosynthesis (Dörr et al., 2014), D-Arg targets cell wall independent pathways, thus explaining the lack of rod-to-sphere induction phenotype in the *V. cholerae mrcA* mutant. In sensitive organisms, like *Caulobacter crescentus* and *Agrobacterium tumefaciens*, D-Arg toxicity was suppressed by mutations in the DnaJ chaperone system and in the phosphate uptake machinery, confirming the different roles that D-amino acids play in bacterial physiology. The reason why D-Arg sensitivity is suppressed by mutations in these pathways remains still unknown, but provides new and interesting research possibilities. It is tempting to speculate about the induction and cross-complementation of different chaperone systems exerted by the anomalous incorporation of D-amino acids into proteins and concomitant alteration of the protein patterns. Chaperone systems might help refold or degrade toxic misfolded proteins. The fact that one type of D-amino acids (e.g., D-Arg) induces such a response and not others (e.g., D-Met) further supports the different mechanisms of action. The role of inorganic phosphate (Pi) in resistance to D-Arg is even more elusive, being Pi a central element of numerous metabolic and regulatory networks.

In addition, the study has shown that the ability to produce D-amino acids is not universally widespread (Alvarez et al., 2018). BsrV orthologs are missing in some Vibrionaceae species, although all tested members of the family (41 species) can grow at high mM concentrations of diverse D-amino acids. Since Vibrio species normally coexist in diverse marine, fresh water and host ecosystems, it is highly possible that cooperative strategies have been established between them to benefit of the secreted D-amino acids as a community. To support this hypothesis, the authors demonstrated that in the presence of L-Arg, a mixture of both BsrV+ (wild-type) and BsrV- (*bsrV* mutant) *V. cholerae* was able to outcompete *C. crescentus*, while BsrV- cells alone could not. The authors propose that D-Arg production could be a public good shared in Vibrio communities, i.e., while some members of the community have specialized and act as D-amino acid producers, non-producer vibrios, “the cheaters,” indirectly benefit from the production of D-amino acids such as D-Arg used to control sensitive bacteria populations.

Given the relatively easy occurrence of suppressor mutations conferring resistance to certain D-amino acids, it seems reasonable that bacteria produce more than one type of amino acid, as these imply (i) divergent mechanisms to attack different targets at the same time, and (ii) the capacity to produce D-amino acids under varying L-amino acids availability.

### Potential Application of D-Amino Acids in Antimicrobial Treatments

Due to their antibiofilm and bactericidal effect, application of D-amino acids is an attractive antimicrobial strategy both alone or in synergy with existing antibiotics. Moreover, combinatory treatments with several D-amino acids can be more effective and prevent the emergence of suppressor mutants, since different D-amino acids target distinct pathways.

A cocktail of D-amino acids efficiently enhanced sublethal concentration of THPS (tetrakis hydroxymethyl phosphonium sulfate), a commonly used antimicrobial reagent used in water treatment processes, in two types of biofilm consortia (Li Y. et al., 2016). However, the D-amino acid mix required for biofilm dispersal might vary depending on the species combinations. In addition, D-amino acids also enhanced the activity of the biocide NALCO7330 (active components: 5-chloro-2-methyl-4-isothiazolin-3-one and 2-methyl-4-isothiazolin-3-one) against biofilm on the steel coupons retrieved from a water cooling tower (Jia et al., 2017).

D-Leu applied to citrus tree leaves alone, or in combination with copper, reduced the number of canker lesions and populations of *Xanthomonas citri* subsp. *citri* (Li and Wang, 2014). Interestingly, D-Leu inhibited biofilm formation in this bacterium, however, genes important for biofilm, chemotaxis and motility were not differentially expressed thereby suggesting a post-transcriptional mechanism of biofilm dissociation. D-Leu foliar application might be a promising strategy to reduce the usage of copper bactericides and avoid copper resistance in xanthomonad populations.

### D-Amino Acids as a Sole Carbon and Nitrogen Source

It is well documented that microorganisms preferentially utilize L-amino acids over D-amino acids (Azúa et al., 2014; Zhang and Sun, 2014). However, D-amino acids have been also found in different environments such as in the soil, lakes, rivers, and oceans (Pollock et al., 1977; Dittmar et al., 2001; Kawasaki and Benner, 2006; Wedyan and Preston, 2008). The ability to utilize D-amino acids might be a beneficial trait for bacteria in case of nutrient scarcity and/or high competition for food resources as bacteria able to grow on D-amino acids as a sole source of carbon and nitrogen were found in these ecosystems (Kubota et al., 2016; Pikuta et al., 2016; Radkov et al., 2016). Interestingly, the Gram positive bacterial strain LZ-22^T^, isolated from moss rhizosphere was able to utilize D-Met, D-Leu, D-His, and D-Val. While both enantiomeric forms of Met and Leu supported growth, only the D-form of His and Val was accepted (Pikuta et al., 2016). The draft genome sequence of LZ-22^T^ revealed that bacterium has more than 30 potentially catabolic dehydrogenases, and a variety of genes associated with racemase and isomerase activities.

Presence of dehydrogenases and other enzymes and metabolic pathways for D-amino acid utilization were reported in various bacteria, such as *P. aeruginosa* (Marshall and Sokatch, 1968), *Escherichia coli* (Raunio et al., 1973; Franklin and Venables, 1976), *Helicobacter pylori* (Tanigawa et al., 2010), *Sinorhizobium meliloti* (Chen et al., 2016), and *Proteus mirabilis* (Xu et al., 2017) among others. In yeasts, oxidative deamination of amino acids is performed by D-amino acid oxidases, which also allows them to use D-amino acids for growth (Simonetta et al., 1989; Yurimoto et al., 2000).

## Bacteria-Host Interactions Regulated By D-Amino Acids

### Role of D-Amino Acids in the Animal Host

D-Amino acids have been also demonstrated to influence important physiological aspects of eukaryotic organisms. Thanks to the development of improved analytical methods, D-amino acids as such as D-Ser, D-Asp, D-Ala, and D-Cys have been found in mammalian tissues (Kiriyama and Nochi, 2016). D-Ser is a neurotransmitter that regulates signaling in the cerebral cortex and is involved in memorization and learning (Hashimoto et al., 1992; Mori and Inoue, 2010). D-Asp is mainly present in the central nervous, neuroendocrine and endocrine systems, being involved in hormone secretion (D’Aniello, 2007; Homma, 2007). The physiological function of other D-amino acids has posed a great interest and is currently being studied by many researchers.

**FIGURE 2:**
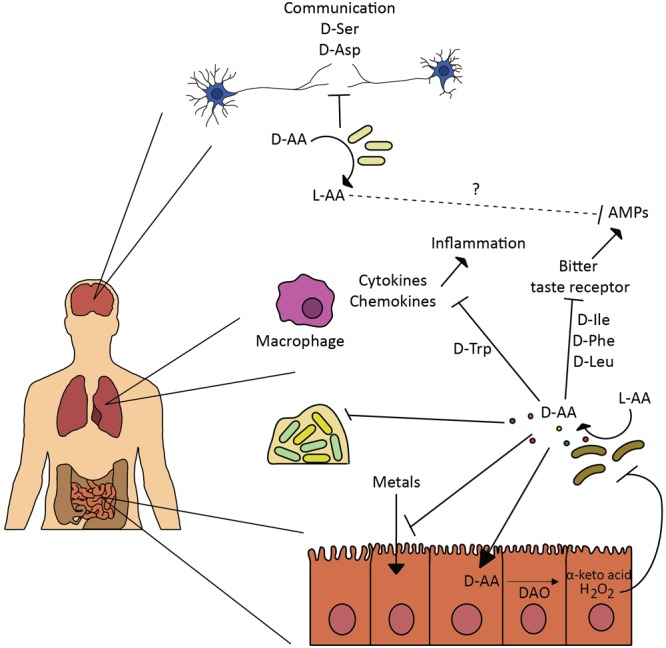
Effect of D-amino acids on human host. Bacteria are involved in various physiological processes in the human body by regulating L- and D-amino acid availability. Such modulation affects neural communication (Kawase et al., 2017), inflammation response induced by cytokines and chemokines (Kepert et al., 2017), production of anti-microbial peptides (AMPs) through inhibition of bitter taste receptors-signaling mechanism (Lee et al., 2017), and metals absorption (Ghssein et al., 2016). Bacteria secrete D-amino acids to inhibit growth and biofilm of competitors (Rasmussen et al., 2000; Lee et al., 2017), in similar fashion bacterial released D-amino acids and interplay with the intestinal D-amino acid oxidase modulate gut homeostasis and microbiota composition (Sasabe et al., 2016).

D-Amino acids not only exhibit a differential role in complex bacterial communities by directly interfering with the different bacteria populations, but hosts and microbes have evolved to interact, and several examples illustrate the great potential of D-amino acids as interkingdom signaling mechanisms (**Figure 2**).

Gut microbiota is composed by a great diversity of bacterial species, some of which, release abundant and diverse D-amino acids in the host (Sasabe et al., 2016). Recently, a study from Waldor lab explored the role of D-amino acids in the gut homeostasis. The intestinal epithelium cells produce a D-amino acid oxidase (DAO), an enzyme that regulates the levels of endogenous D-amino acid by converting them to α-keto acids and H_2_O_2_. The release of H_2_O_2_ to the gut lumen has a toxic effect on sensitive bacterial populations and thus is an important host defense factor (Nathan and Cunningham-Bussel, 2013). This effect was totally dependent on the production of D-amino acids (D-Ala, D-Asp, D-Glu, and D-Pro) by the commensal microbiota, since no D-amino acids were detected in germ-free mice, while abundant amounts of L-amino acids were detected (even greater levels in germ-free mice, likely due to their consumption by the gut microbiota). *In vitro* studies with DAO and D-amino acids resulted in reduced viability of diverse enteric pathogens tested, including *V. cholerae*. Furthermore, this DAO-induced toxicity was attenuated by the catalase activity. Strains deficient in D-amino acids production proved to be better intestinal colonizers than wild-type *V. cholerae*, a difference that was attenuated in DAO mutant mice. The work raised the possibility that DAO could play a role in the protection of the mucosal surface and concluded that the gut microbiota composition can be modulated by released microbial D-amino acids and their interplay with the intestinal DAO. The authors also suggested that additional mechanisms might contribute to the altered microbiota in DAO null mutants, which is consistent with the direct effect of some D-amino acids on the viability of other bacteria.

Another study revealed that the gut microbiota can be an important regulator of amino acid metabolism (Kawase et al., 2017). The microbiome can modulate the amount of amino acids found in the blood and brain of the host, since gut microbiota secretes and metabolizes D-amino acids, influencing their absorption and thus, stimulating the immunological system. In a comparative experiment, the production of D-Ser by the host was inhibited by the gut microbiota: D-Ser concentration in the plasma was higher in germ free than in control mice, although the concentration of L-Ser remained fairly constant. By altering the D-Ser metabolism, the gut microbiota can regulate neurological diseases in the host brain.

Not only the nervous system is influenced by the gut microbiota. Kepert et al. (2017) demonstrated the interplay between the production of D-Trp by probiotic bacteria and allergic airway disease. A thorough screening for bioactive probiotic metabolites revealed the immunomodulatory role of D-Trp. Only D-Trp produced by different *Lactobacillus* species showed bioactivity and decreased the production of TH2 cytokines and chemokines, preventing the development of allergic airway inflammation and hyper-responsiveness. An altered gut microbiota can hence impact the gut immunity directly or indirectly through the release of D-Trp. Bacterial diversity analysis revealed a reduced community richness on mice with allergic airway disease. Interestingly, supplementation with D-Trp led to an increased bacterial diversity, similar to that of healthy mice.

A last example illustrating the interkingdom signaling in the airway mediated by bacterial D-amino acids and the mammalian sweet taste receptor is presented in the study by Lee et al. (2017) about the activation of the sweet taste receptors by D-amino acids and the effects on the airway epithelial innate immune response. The study explored the production of D-Ile, D-Phe, and D-Leu by respiratory isolates of *Staphylococcus* species. These specific D-amino acids activated the sweet taste receptors in the digestive and the upper respiratory tract, both inhibiting the bitter taste receptors-signaling mechanism and defensin secretion in sinonasal cells. Release of antimicrobial peptides (AMPs), like β-defensins, depended on the activation of the bitter taste receptors in the epithelial cells, so bacteria, such as *S. aureus*, have devised a mechanism to suppress innate immune responses and minimize their own death, thus protecting themselves from eradication and promoting the colonization of the respiratory tract. It remains to be explored whether this mechanism provides a host benefit *in vivo*, like the attenuation of the immune responses against commensal bacteria, or whether this is an evasion mechanism by pathogenic bacteria.

Additionally, released D-amino acids secreted by non-pathogenic components of the normal respiratory flora (Rasmussen et al., 2000) are used to prevent the growth of competing bacteria in the airways, such as *P. aeruginosa*. The researchers confirmed that opportunistic bacteria such as *S. aureus* also take advantage by suppressing *P. aeruginosa* virulence through the secretion of these D-amino acids, which interfere with its biofilm formation capacity (Lee et al., 2017).

### D-Amino Acids as Building Blocks of Proteins and Antimicrobial Peptides

D-Amino acids are also building blocks of certain compounds used by both bacteria and host cells to combat each other or survive under stressful conditions.

The presence of D-amino acids as building blocks of peptides and proteins dates back to the late 20s (Morizawa, 1927), when octopine, a derivative of L-arginine and D-alanine produced by octopuses was first described. At first it was believed that only the L-configuration was allowed in the structure of peptides and proteins, however, numerous D-amino acid containing peptides have been described since the 80s (numerous examples of eukaryotic and bacterial peptides are summarized in Cava et al., 2011b), when D-containing residues were reported in frog skin opioid peptides (Yamashiro et al., 1983; Amiche et al., 1989). Soon it was demonstrated that most organisms are capable of producing diastereomeric peptides and proteins (Ollivaux et al., 2014). The presence of D-amino acids in the peptide structure generally enhances its activity and stability, and it can play a key role for receptor recognition (Li H. et al., 2016). This is one of the main reasons why D-amino acids in host defense peptides (HDP) improve the efficacy of the next generation of broad spectrum therapeutic agents.

Antimicrobial peptides (AMPs) or HDP are efficient and versatile immune molecules bioactive against all types of pathogens, including bacteria, viruses, fungi, parasites even cancerous cells (Wang et al., 2010a, 2014; Hilchie et al., 2011; Lynn et al., 2011; Hong et al., 2014). AMPs are short peptides, between 12 and 50 residues, produced by all living organisms and they present not only antimicrobial activity but also immunomodulatory functions. Their mechanism of action can be diverse: (i) AMPs can bind and disrupt the membrane structural integrity, through pore formation or detergent like mechanisms (Bahar and Ren, 2013; Wang, 2014); (ii) AMPs disperse biofilms by reducing the adhesion to surfaces, killing of embedded bacteria or interfering with the metabolic pathways involved in biofilm formation (de la Fuente-Nunez et al., 2015; Segev-Zarko et al., 2015); (iii) AMPs influence inflammation and recruitment of dendritic cells, hence modulating the immune response (Tani et al., 2000; Hubert et al., 2007; Wang et al., 2010b; Lee et al., 2011); (iv) some AMPs can induce apoptosis (Mader et al., 2005; Kim et al., 2012).

So far, no natural AMPs composed only of D-amino acids have been described. Some antibiotics like penicillins and cephalosporins, contain a D-Val moiety and a cycloserine derived from D-Ser (Baldwin and Schofield, 1992; Schofield et al., 1997). Other more complex peptide antibiotics (gramicidin, actinomycin, bacitracin, or polymyxin) are assembled in a stepwise fashion by the action of specific peptide synthetases that catalyze individual reactions (Ollivaux et al., 2014). Gramicidin was the first antibiotic peptide to be used clinically (Gall and Konashev, 2001; Kelkar and Chattopadhyay, 2007). These molecules produced by *Bacillus brevis* alternate L- and D-amino acids in their sequence and act through the formation of ion channels that disrupt cell membranes (Hladky and Haydon, 1972; Kelkar and Chattopadhyay, 2007). *B. brevis* produces other AMPs such as gratisin GR (Tamaki et al., 2011) and tyrocidines (Loll et al., 2014), which also act through membrane disruption.

To date, the most extended explanation for D-amino acid presence in ribosomally synthetized proteins is through the post-translational modification of L-amino acid peptides/proteins, since there is no *in vivo* evidence that ribosomes can incorporate D-amino acids to the peptide chain or that the L-residue is excised and immediately substituted by its D-counterpart (Heck et al., 1996; Torres et al., 2006; Ollivaux et al., 2014). This is the case of the lantibiotics, bacteriocins produced by Gram-positive bacteria (Skaugen et al., 1994; Ryan et al., 1999; Cotter et al., 2005). Although some studies demonstrate that tRNAs can be charged with D-amino acids *in vitro*, their incorporation into peptides/proteins *in vivo* requires the absence of the corresponding D-amino acid-tRNA deacylase (Soutourina et al., 2000; Goto et al., 2008; Leiman et al., 2013).

It is tempting to speculate about the effect on antimicrobial production of bacteria harboring broad spectrum racemases that can modulate the availability of D-amino acids in the media. It is plausible that such racemases could be produced as defensive mechanisms by reducing the substrate availability and hence the biosynthesis flow of antimicrobial compounds.

### D-Amino Acids Role in Metal Scavenging

Recently, a novel metal scavenging molecule named staphylopine has been discovered to be produced by *S. aureus* (Ghssein et al., 2016). Since metals are essential elements for all organisms, the phenomenon known as nutritional immunity (Corbin et al., 2008; Hood and Skaar, 2012), the process by which a host organism sequesters trace minerals to limit the pathogenicity during infection, stands out as a strategy to combat bacterial infections. However, different metal uptake mechanisms have also been devised by the invading bacteria. Staphylopine is synthetized by combination of D-His, amino butyrate and pyruvate, it is then released to the extracellular media where it traps the target metals, including nickel, zinc, cobalt, copper and iron, and finally an import system recovers the complex, abolishing the metal starvation state imposed by the host. As expected, *S. aureus* cells deficient in staphylopine production exhibited reduced virulence and fitness during host infection.

Other bacteria and plants also use His or other amino acids such as nicotianamine in plants for the synthesis of metal chelators (Schauer et al., 2007; Curie et al., 2009; Walker and Waters, 2011; Lebrette et al., 2015). The fact that different bacteria produce and release a wide set of D-amino acids to the extracellular media raises the question whether these molecules could also be playing a key role in the synthesis of other metallophores with different metals affinities. Therefore, production of such molecules might provide a competitive advantage against other bacteria within the same niche. It would be interesting to investigate whether metal homeostasis, bacterial fitness and population dynamics in the host is influenced by microbial D-amino acid production.

### Role of D-Amino Acids in the Plant Development and Health

**FIGURE 3:**
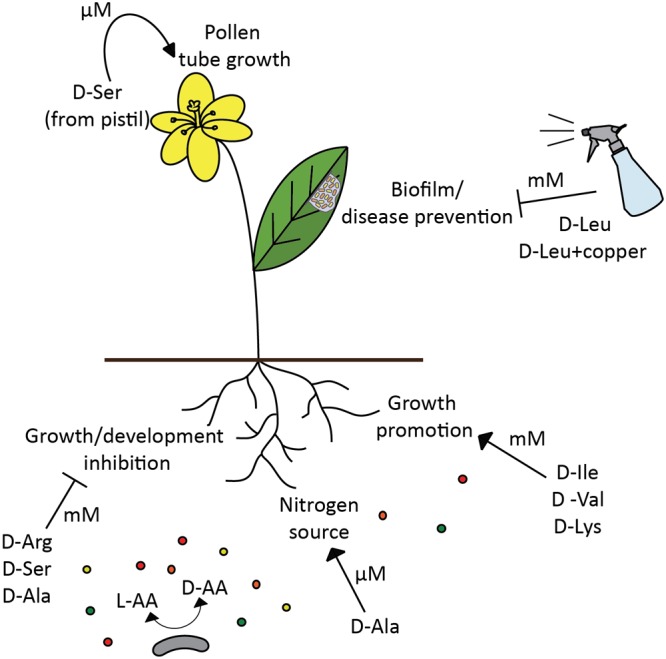
D-amino acids modulate plant development and health. Depending on D-amino acid concentration (μM vs. mM), the effect of the same amino acid (D-Ser) in the plant can be either positive, regulation of pollen tube development (Michard et al., 2011), or detrimental, plant growth inhibition (Forsum et al., 2008). However, different D-amino acids not only inhibit plant growth (Erikson et al., 2004; Forsum et al., 2008), but also can promote it (Erikson et al., 2004; Chen et al., 2010), and be assimilated as a nitrogen source (Hill et al., 2011). Certain D-amino acids have a potential to be used for disease prevention in plants (Li and Wang, 2014). Broad spectrum racemase bacteria are likely to be an important modulator of D-amino acids availability in the soil, thus affecting various processes in plants and selecting plant associated bacterial populations.

It was documented that plants are readily able to uptake D-amino acids from the soil (Aldag and Young, 1970; Svennerstam et al., 2007; Forsum et al., 2008; Vranova et al., 2012), nevertheless, the physiological role of these molecules in the plant is still far from being clear. For a long time, plant growth inhibition by certain D-amino acids, and slow degradation of D-amino acids by plants neglected the possibility that D-amino acids could be serving as nitrogen source or play a role as important regulatory molecules (**Figure 3**) (Erikson et al., 2004; Svennerstam et al., 2007; Forsum et al., 2008; Näsholm et al., 2009). D-Ser (0.5 mM), D-Ala (1 mM), and D-Arg (0.75 mM) were shown to have a strong inhibition effect on the growth of *Arabidopsis* (Erikson et al., 2004; Forsum et al., 2008). However, not all the D-amino acids have detrimental effect on plants, and some D-amino acids can even promote plant growth, such as 5 mM D-Ile and 1 mM D-Val enhanced *Arabidopsis* growth (Erikson et al., 2004), while 2 mM D-Lys, but not L-Lys, was efficient in promoting growth of both *Arabidopsis* and tobacco (Chen et al., 2010). In addition, plants respond differently to the presence of D-amino acid in growth medium and foliar application, and submillimolar less toxic concentrations might be a more realistic representation of physiological concentration found in the soil in nature (Erikson et al., 2004; Chen et al., 2010; Gördes et al., 2011).

There is also growing evidence that D-amino acids can be both produced and metabolized by plants, since D-amino acid synthesizing and degrading enzymes, such as racemases, D-amino acid aminotransferases or D-amino acid oxidases, have been described in different plants (Fujitani et al., 2006, 2007; Ono et al., 2006; Funakoshi et al., 2008; Gholizadeh and Kohnehrouz, 2009). Moreover, Hill et al. (2011) showed that D-Ala can be taken up and assimilated by wheat from the solution of mixed nitrogen forms, where D-Ala uptake was five-fold faster than NO_3_-. This finding opposes the idea that D-amino acids are irrelevant for plants and serve only as phytotoxic molecules. Michard et al. (2011) brought yet another argument for the role of D-amino acids as important modulators of plant development. Their study has shown that D-Ser influences pollen tube development in *Arabidopsis* and tobacco, and D-serine racemase is important for D-Ser mediated signal transduction (Michard et al., 2011).

## Concluding Remarks

Given the great importance of D-amino acids, the bacteria that produce them play a key role in the regulation of L- and D-amino acids availability in various environments. Summarizing information about the activity of bacterial secreted D-amino acids, their autocrine effect on producer organisms as well as their impact on other microbes or hosts suggests that we cannot think of D-amino acids as just one single type of molecule, but rather as specific effector with unique biological activities. Therefore, coming research efforts will be heading to figure out the mechanism of each D-amino acid in a specific organism and their ecological significance.

## Author Contributions

All authors listed have made a substantial, direct and intellectual contribution to the work, and approved it for publication.

## Conflict of Interest Statement

The authors declare that the research was conducted in the absence of any commercial or financial relationships that could be construed as a potential conflict of interest.
